# Ultrastructure of Antennal Sensory Organs in Nine Flesh Flies (Diptera: Sarcophagidae): New Insight into the Definition of Family Sarcophagidae

**DOI:** 10.3390/insects13070602

**Published:** 2022-06-30

**Authors:** Wentian Xu, Genting Liu, Qike Wang, Liping Yan, Xianhui Liu, Xinyu Li, Thomas Pape, Dong Zhang

**Affiliations:** 1 School of Ecology and Nature Conservation, Beijing Forestry University, Qinghua East Road 35, Beijing 100083, China; xuwt720@bjfu.edu.cn (W.X.); gentingl@student.unimelb.edu.au (G.L.); yanlp@bjfu.edu.cn (L.Y.); lixinyu91@cau.edu.cn (X.L.); 2 School of BioSciences, University of Melbourne, Melbourne, VIC 3010, Australia; wangqike123@gmail.com; 3 Department of Entomology and Nematology, University of California Davis, Davis, CA 95616, USA; xhfliu@ucdavis.edu; 4 Natural History Museum of Denmark, Science Faculty, University of Copenhagen, 2100 Copenhagen, Denmark; tpape@snm.ku.dk

**Keywords:** antennal sensilla, comparative morphology, flesh flies, Miltogramminae, Paramacronychiinae, phylogeny, Sarcophaginae

## Abstract

**Simple Summary:**

The antennal sensilla of species in all three subfamilies of Sarcophagidae are studied for the first time via scanning electron microscopy. The morphology, density, and distribution of each type are described for each species. A total of eight types of antennal sensilla (chaetic sensilla, setiferous plaques, pedicellar buttons, trichoid sensilla, basiconic sensilla, coeloconic sensilla, clavate sensilla, bottle-shaped sensilla) and sensory pits are found in both sexes. The existence of bottle-shaped sensilla in the sensory pits in all three subfamilies of the sarcophagid species suggests a potential synapomorphy of sarcophagids and a new morphological diagnosis character of the family Sarcophagidae.

**Abstract:**

The antennae are the main olfactory organ of flies, playing key roles in their survival and the success of all life stages. Antennal ultrastructural morphology has been well described in the representative species of most calyptrate families, yet only a few studies have focused on Sarcophagidae species, those with ecological and medical relevance. Antennal morphology and the types, shapes, distribution, and density of the antennal sensilla of nine Sarcophagidae species are studied in detail with scanning electron microscopy, including Miltogramminae: *Metopia campestris* (Fallén) and *Mesomelena mesomelaena* (Loew), Paramacronychiinae: *Agria mihalyii* (Rohdendorf & Verves), *Wohlfahrtia bella* (Macquart), and *W*. *magnifica* (Schiner); Sarcophaginae: *Sarcophaga* (*Parasarcophaga*) *albiceps* Meigen, *S*. (*Bercaea*) *africa* (Wiedemann), *S*. (*Boettcherisca*) *peregrina* (Robineau-Desvoidy), and *S*. (*Liosarcophaga*) *portschinskyi* (Rohdendorf), covering all three subfamilies of this family. The morphology of the three segments of the antennae has been described. The scape has only one type of chaetic sensilla, while three subtypes of chaetic sensilla were detected on the pedicel. The postpedicel has four types of sensilla: trichoid sensilla, coeloconic sensilla, clavate sensilla, and three subtypes of basiconic sensilla. Bottle-shaped sensilla were observed in sensory pits on the postpedicel in all nine species. These sensilla have not been discovered in other calyptrate species, suggesting that they are a potential sarcophagid synapomorphy.

## 1. Introduction

The Sarcophagidae (flesh flies) is a widespread family that consists of more than 3000 species [1,2]. Flesh flies are classified into three recognized subfamilies: Miltogramminae, Paramacronychiinae, and Sarcophaginae [1,3,4,5]. They have diverse habits and play important roles in ecosystems as pollinators and decomposers. Several representatives of these three subfamilies are strongly synanthropic, including a few species reported as facultative or obligate myiasis producers, causing traumatic myiasis in humans, livestock, and wildlife [6,7,8,9,10].

The subfamily Miltogramminae are known as “satellite flies” since many are kleptoparasitic in the nests of solitary wasps and bees (e.g., Apoidea; Sphecidae) [11,12]. The first instar larvae of some miltogrammine species (e.g., *Eumacronychia persolla* Reinhard, *Phylloteles pictipennis* Loew, *Mesomelena mesomelaena* (Loew)) can burrow into the soil and reach deeply buried animal remains [13,14]. The Paramacronychiinae are flies with diverse feeding habits such as predators, parasites, or parasitoids of insects. In the genus *Agria* Robineau-Desvoidy, the larvae of *A*. *mihalyii* (Rohdendorf & Verves) are parasitoids of the last instar larvae and pupae of Lepidoptera [15,16,17]. The larvae of several paramacronychiine flies are responsible for myiasis in humans or other mammals (e.g., *Wohlfahrtia* spp.) [10,18,19]. The species of Sarcophaginae exhibit a high-level diversity of feeding habits, such as agents of myiasis, predators, parasitoids of various insects, and decomposers of organic matter [20].

As the most important olfactory organ of flies, the antennae provide insects with critical information about their environment, playing a significant role in key activities such as foraging, mate recognition, and locating breeding substrates [21,22,23,24,25]. Strong selection pressure has shaped the morphology of insect antennae [26,27,28]. Thus, as an important phylogenetic character, the antennae of flies have evolved into an amazing diversity of shapes and sizes and are richly endowed with antennal sensilla of various types, numbers, and distribution [29,30].

Several studies have showed the importance of discovering new morphological characters in the phylogenetic studies of Diptera, even in the era of molecular phylogeny [31]. The comprehensive description of the antennae and antennal sensilla ultrastructure has contributed to the systematics of Diptera. For instance, they provide taxonomic information for Muscidae [32] and indicate the evolutionary trends of Ceratopogonidae [33]. Flesh flies are one of the most common and most diverse families of flies, but surprisingly, only a few external morphological characteristics can be used to define this taxon. So far, only the shortened bacilliform sclerites in the male terminalia and abdominal sternites without alpha setae [1] are external characteristics of this family. Although the phylogenic monophyly of Sarcophagidae has been extensively corroborated [34,35], the lack of clear morphological diagnosis imposes difficulties in taxonomic studies. Furthermore, studies on the antennal ultrastructure of sarcophagid flies are limited in comparison to its diversity [30,36,37,38,39], and no comparative study covering all of the subfamilies of Sarcophagidae has been published.

In Sarcophagidae, the antennal ultrastructure has been examined in *Wohlfahrtia nuba* (Wiedemann) [40], *S*. *bullata* Parker [41], *Sarcophaga babiyari* (Lehrer) [38], *Sarcophaga dux* Thomson [30], and *Sarcophaga tibialis* Macquart [39]. Greenberg and Ash [42] examined the morphology of setiferous plaques in *Boettcheria cimbicis* (Townsend), *Sarcophaga aldrichi* Parker, and *Sarcophaga bullata*. Optical and transmission electron microscopy studies of the sensory organs on the antennal postpedicel were carried out in *Sarcophaga carnaria* (Linnaeus) [36] and in *Sarcophaga argyrostoma* (Robineau-Desvoidy) [37].

Here, we describe the morphology of the antennae and antennal sensilla of nine species from five genera covering all three subfamilies of Sarcophagidae (Miltogramminae: *M*. *campestris* (Fallén) and *M*. *mesomelaena*; Paramacronychiinae: *A*. *mihalyi*, *W*. *bella* (Macquart), and *W*. *magnifica* (Schiner); and Sarcophaginae: *S*. (*Parasarcophaga*) *albiceps* Meigen, *S*. (*Bercaea*) *africa* (Wiedemann), *S*. (*Boettcherisca*) *peregrina* (Robineau-Desvoidy), and *S*. (*Liosarcophaga*) *portschinskyi* (Rohdendorf)) to comparatively analyze antennal sensilla in Sarcophagidae and to discover shared characteristics of the antennal sensilla among the subfamilies.

## 2. Materials and Methods

Among different families in the calyptrate species, where both males and females have been studied, both sexes have morphologically similar antennae and the same types of sensilla [23,24,25,29,30,32,39]. Here, we examined nine species from three subfamilies. The adults of *M*. *mesomelaena* (3♂, 3♀), *S*. *portschinskyi* (3♂, 3♀), *W*. *bella* (3♂, 3♀), and *W*. *magnifica* (3♂) were collected in Mount Kalamaili Ungulate Nature Reserve, Xinjiang; *A*. *mihalyii* (3♂), *M*. *campestris* (3♂), *S*. *albiceps* (3♂), *S*. *africa* (3♂), and *S*. *peregrina* (3♂) were collected on the campus of Beijing Forestry University and the Beijing Songshan National Nature Reserve. All of the specimens were pinned and air-dried on site. The examined specimens were deposited in Beijing Forestry University. 

The morphology of the antennae was examined with a SteREO Discovery.V12 stereoscopic microscope (ZEISS Corp., Oberkochen, Germany). The head of each specimen was cut off and rehydrated in phosphate-buffered saline (pH 7.4) for 30 min. The antennae were then dissected from the head and rinsed with detergent in a sonicator. To examine the sensilla in the sensory pits, the antennae from two specimens of each species were cut longitudinally with a razor blade [43]. After dehydration in a graded ethanol series, the antennae were mounted in the proper orientation on round aluminum stubs (15 mm in radius, 6 mm in height) with conductive adhesive and left in a desiccator to dry for 24 h. Then, the samples were coated with gold and observed using HITACHI S3400 scanning electron microscope (Hitachi Corp., Tokyo, Japan). Micrographs were taken at various magnifications, showing the antenna and antennal sensilla. 

Dimensions, including the length, basal diameter, tip diameter (clavate sensilla only), basal swelling degree (maximum diameter of the ‘neck’ of the bottle/maximum diameter of the bottle half, bottle-shaped sensilla only), and density of the antennal sensilla, were then measured. The length of each single sensillum was measured and defined as the proximal rim to the tip (*n* = 10 sensilla used per sample). The densities of each type of sensillum were measured by taking quadrates (each representing 21 × 21 µm^2^) from the distal, median, and proximal parts of the antenna on both sides, and ten quadrates were taken from each part of each side [44,45,46]. The terminology used to describe the antennal morphology and the classification of sensilla types followed Cumming and Wood [47].

## 3. Results

### 3.1. General Description of the Antennal Morphology of the Nine Species of Sarcophagidae

**Figure 1:**
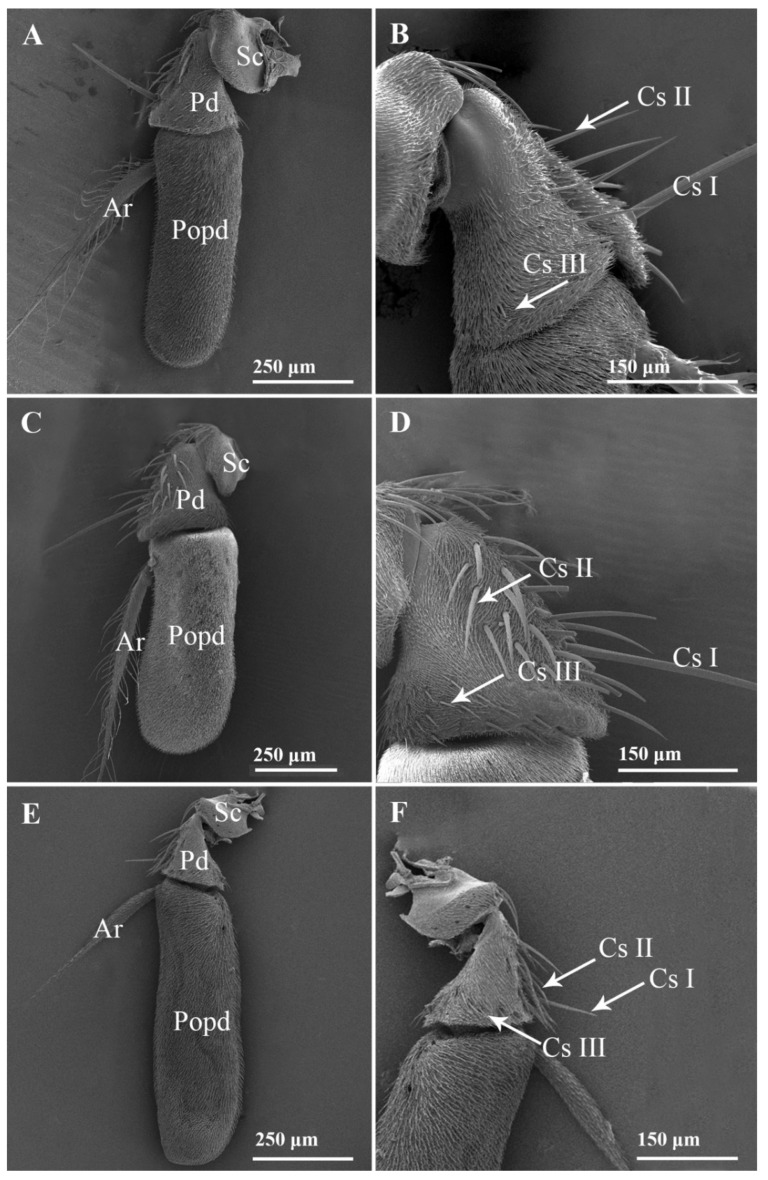
Features on the antennae of male *Sarcophaga portschinskyi*, *Agria mihalyii*, and *Metopia campestris*. (**A**) *S*. *portschinskyi*, (**C**) *A*. *mihalyii*, and (**E**) *M*. *campestris* antenna, showing the anterior surface; (**B**) *S*. *albiceps*, (**D**) *A*. *mihalyii*, and (**F**) *M*. *campestris*, showing three subtypes of mechanoreceptors on the antennal pedicel. Abbreviations: Cs I (subtype I chaetic sensilla); Cs II (subtype II chaetic sensilla); Cs III (subtype III chaetic sensilla); Ar (arista); Popd (postpedicel); Pd (pedicel); Sc (scape).

Like other calyptrate, each of the nine species of Sarcophagidae in this study bears a pair of aristate antennae consisting of three segments: a proximal scape (Sc), a pedicel (Pd), and a distal flagellum composed of a postpedicel (Popd) with an arista (Ar) (Figure 1).The scape is the most proximal and shortest segment, directly attached to the head capsule; the pedicel is the second segment of the antenna, longer than the scape; the postpedicel is the longest and most prominent segment of the antenna, where most of the sensilla are located. The postpedicel can be divided into two sides: an anterior surface and a posterior surface. The arista is attached to the posterior surface of the postpedicel.

#### 3.1.1. Scape and Pedicel

The scape is densely covered with microtrichia, with only few chaetic sensilla characterized as long mane-like sensilla fitted tightly in slightly elevated smooth sockets and longitudinal grooves on the surface and tapered to an acute tip.

The pedicel is covered with microtrichia. Compared to the scape, more chaetic sensilla are found on the surface of the pedicel, which varies in length and can be divided into three subtypes (Cs I, Cs II, and Cs III) (Figure 1). Usually, one longer Cs I can be found on the pedicel, located near the distal region. Cs II are shorter, mainly distributed near the pedicellar cleft. Cs III are the shortest, mainly located in the distal region. The chaetic sensilla on the pedicel are relatively straight, with clusters of microtrichia distributed along the outer edge of the base.

**Figure 2:**
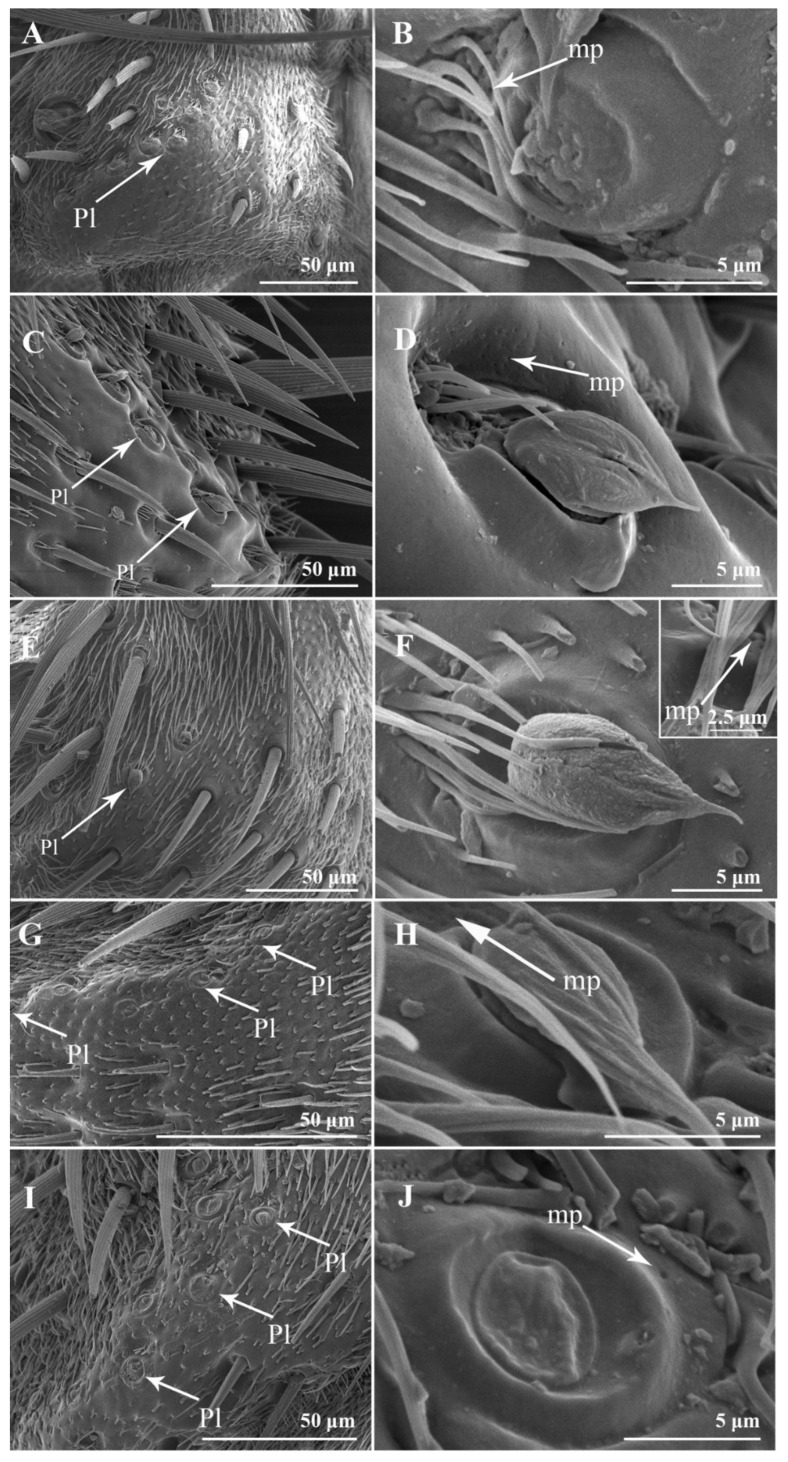
The setiferous plaques on the antennal pedicel of male *S. albiceps*, *S. africa*, *S. peregrina*, *W. mag-nifica*, and *A. mihalyii*. (**A**,**B**) *S. albiceps*, (**C**,**D**) *S. africa*, (**E**,**F**) *S. peregrina*, (**G**,**H**) *W. magnifica*, and (**I**,**J**) *A. mihalyii*. Abbreviations: mp (micropore); Pl (setiferous plaque).

**Figure 3:**
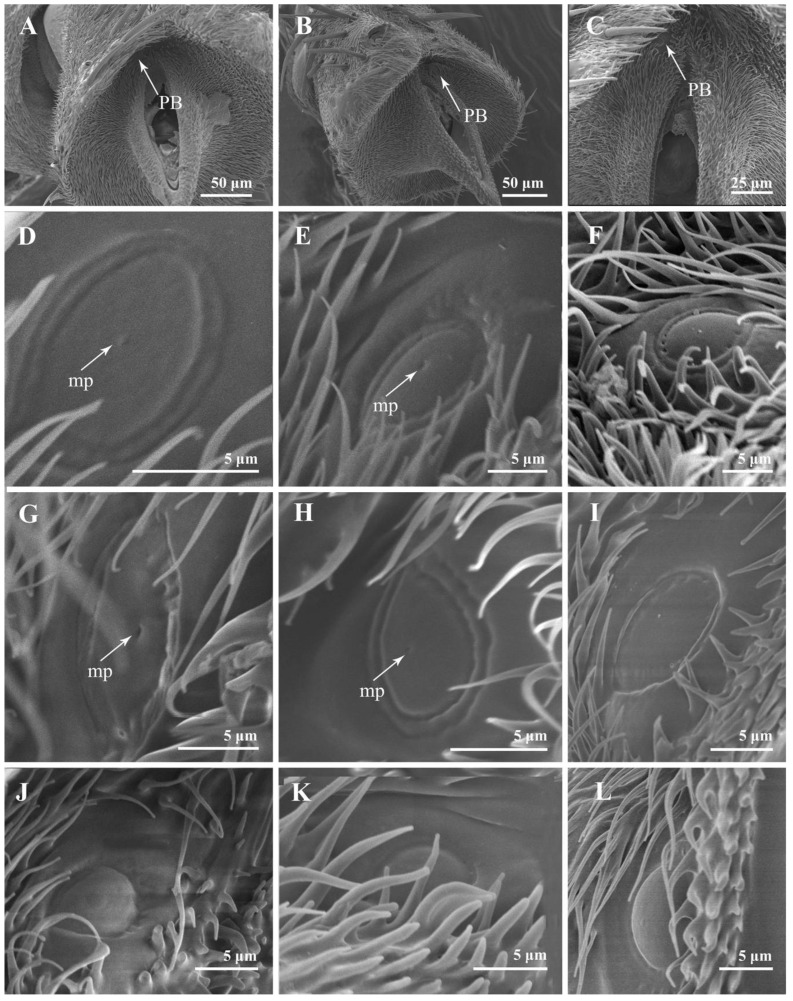
The pedicellar button on the antennal pedicel of nine sarcophagid species. (**A**–**C**) *S*. *albiceps*, *S*. *portschinskyi*, and *W*. *magnifica* after the removal of postpedicel, showing the position of the pedicellar button; (**D**) PB of *S*. *albiceps*; (**E**) PB of *S*. *portschinskyi*; (**F**) PB of *W*. *magnifica*; (**G**) PB of *S*. *africa*; (**H**) PB of *S*. *peregrina*; (**I**) PB of *W*. *bella*; (**J**) PB of *A*. *mihalyii*; (**K**) PB of *M*. *campestris*; (**L**) PB of *M*. *mesomelaena*. Abbreviations: mp (micropore); PB (pedicellar button).

Four to nine setiferous plaques (Pl) are distributed in a slightly elevated and smooth area between Cs II and Cs III on the pedicel (Figure 2A,C,E,G,I). Each setiferous plaque contains a bulbous seta with a sharply abrupt tip slightly extended to an obtuse base that is inserted in a socket with an elevated rim (the bulbous setae are lost, probably due to age, in Figure 2B,J), with a cluster of microtrichia and few micropores at the base (Figure 2B,D,F,H,J). One pedicellar button (PB) located within the dorsal recess and near the pedicellar cleft can be discovered after separating the pedicel and the postpedicel (Figure 3). It is located in the smooth, bare area among the microtrichia and consists of a circular, centrally perforated dome with a slightly convex ring. In *S*. *albiceps*, *S*. *africa*, *S*. *peregrina*, and *S*. *portschinskyi*, one micropore was identified at the center of the pedicellar button (Figure 3D,E,G,H).

#### 3.1.2. Postpedicel

**Figure 4:**
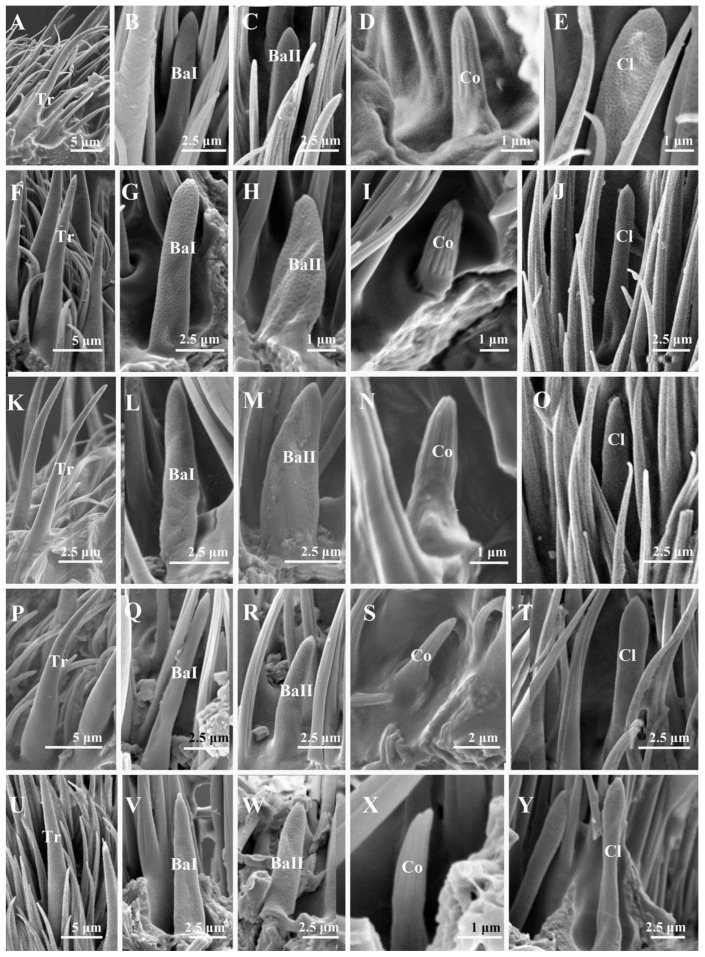
Trichoid sensilla, basiconic sensilla, coeloconic sensilla, and clavate sensilla on the antennal postpedicel of male sarcophagid species. (**A**–**E**) *S. albiceps*; (**F**–**J**) *S. portschinskyi*; (**K**–**O**) *S. africa*; (**P**–**T**) *S. peregrina*; (**U**–**Y**) *W. magnifica*. Abbreviations: Co (coeloconic sensilla); Cl (clavate sensilla); Ba I (subtype I basiconic sensilla); Ba II (subtype II basiconic sensilla); Tr (trichoid sensilla).

**Figure 5:**
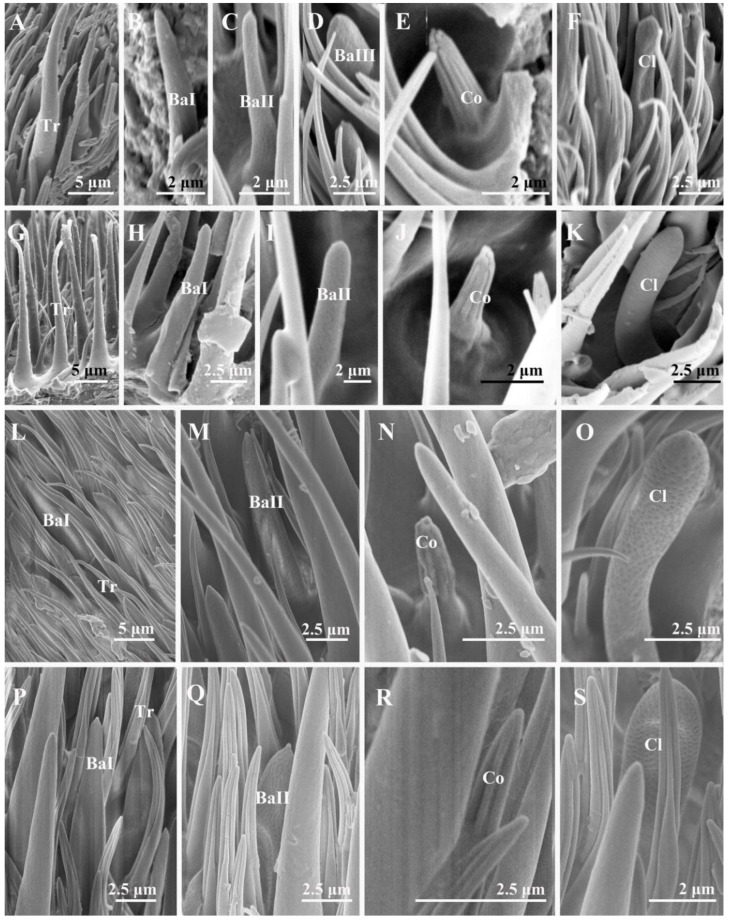
Trichoid sensilla, basiconic sensilla, coeloconic sensilla, and clavate sensilla on the antennal postpedicel of male sarcophagid species. (**A**–**F**) *A*. *mihalyii*; (**G**–**K**) *M*. *campestris*; (**L**–**O**) *M*. *mesomelaena*; (**P**–**S**) *W*. *bella*. Abbreviations: Co (coeloconic sensilla); Cl (clavate sensilla); Ba I (subtype I basiconic sensilla); Ba II (subtype II basiconic sensilla); Ba III (subtype III basiconic sensilla); Tr (trichoid sensilla).

**Figure 6:**
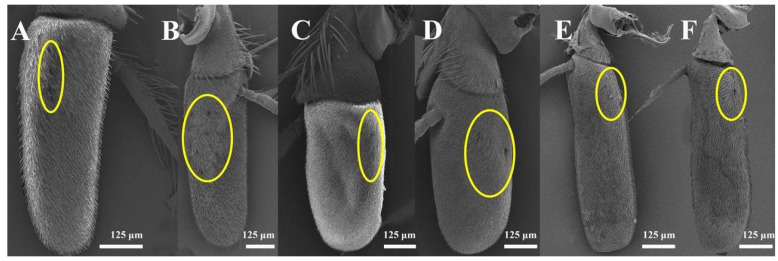
The sensory pits on the postpedicel of nine sarcophagid species. (**A**) *S*. *albiceps*, dorsal view; (**B**) *S*. *albiceps*, ventral view; (**C**) *W*. *magnifica*, dorsal view; (**D**) *W*. *magnifica*, ventral view; (**E**) *M*. *campestris*, ventral view; (**F**) *M*. *campestris*, dorsal view. Yellow circle marks area containing one or more sensory pits.

**Figure 7:**
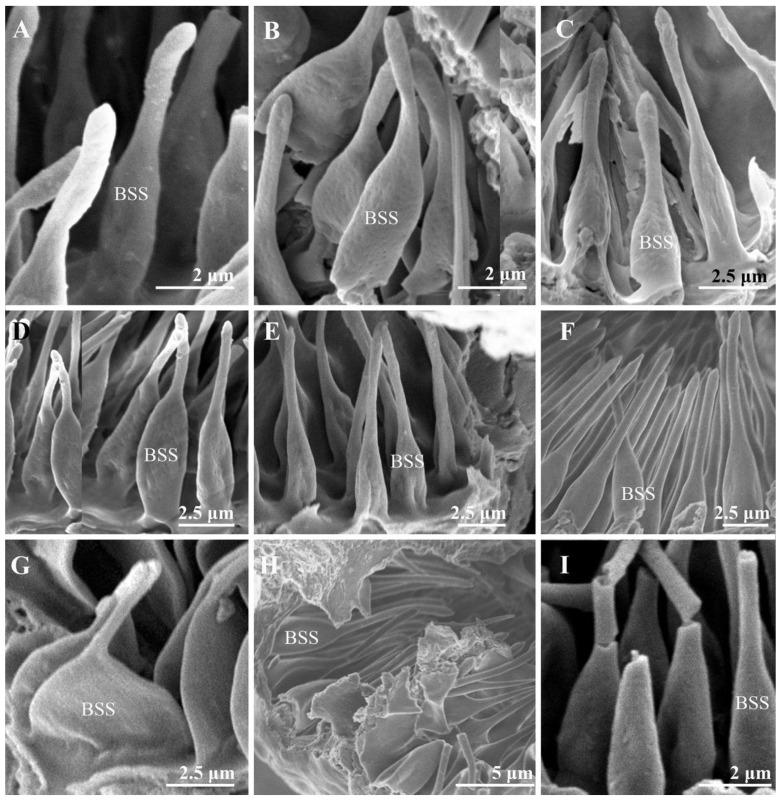
The bottle-shaped sensilla in the sensory pits on the antennal postpedicel of nine male sarcophagid species. (**A**) *S*. *albiceps*; (**B**) *S*. *portschinskyi*; (**C**) *S*. *africa*; (**D**) *S*. *peregrina*; (**E**) *W*. *magnifica*; (**F**) *W*. *bella*; (**G**) *A*. *mihalyii*; (**H**) *M*. *mesomelaena*; (**I**) *M*. *campestris*. Abbreviations: BSS (bottle-shaped sensilla).

**Figure 8:**
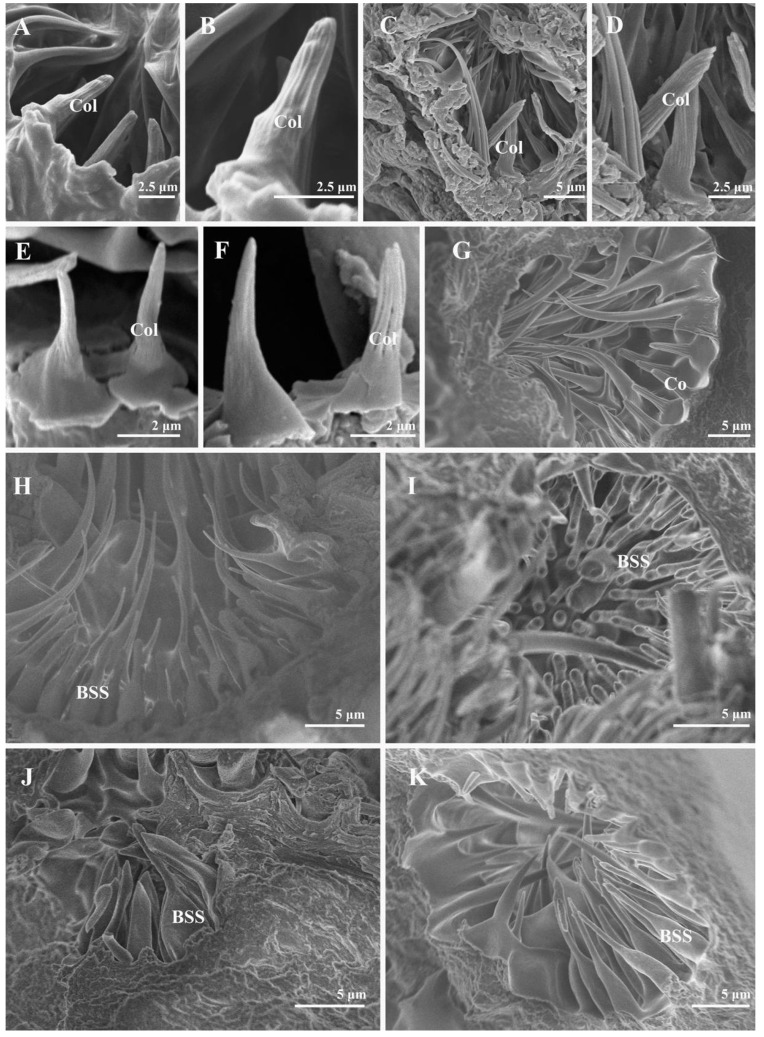
The sensilla in the sensory pits of male and female in sarcophagid species. (**A**,**B**) male of *S*. *albiceps*; (**C**,**D**) male of *S*. *peregrina*; (**E**,**F**) male of *W*. *magnifica*; (**G**) female of *W*. *bella*; (**H**,**I**) female of *S*. *portschinskyi*; (**J**) female of *M*. *mesomelaena*; (**K**) female of *W*. *bella*. Abbreviations: BSS (bottle-shaped sensilla); Co (coeloconic sensilla); Col (coeloconic-like sensilla).

The postpedicel is the most important antenna segment, with the largest number and greatest diversity of sensilla types attached. The entire postpedicel surface is covered with dense microtrichia, and four types of sensilla (Figure 4 and Figure 5) can be found on the surface in total: trichoid sensilla (Tr), basiconic sensilla (Ba, including subtypes I, II, and III), coeloconic sensilla (Co), and clavate sensilla (Cl). A number of sensory pits are also present on the postpedicel (Figure 6). Clusters of coeloconic-like sensilla (Col) and bottle-shaped sensilla (BSS) were discovered on the inner surface of the sensory pits (SP) (Figure 7 and Figure 8). No sensilla were detected on the arista.

### 3.2. Sensilla and Sensory Pits on the Postpedicel

The male specimens were examined in all nine species, with the addition of the female specimens of three species. The types of sensilla were the same for both sexes, but a higher number of sensory pits on the postpedicel were discovered in *S*. *portschinskyi*, *W*. *bella*, and *M*. *mesomelaena* (Table 1).

#### 3.2.1. Trichoid Sensilla

Trichoid sensilla (Tr) are the longest and the most numerous among the four types of sensilla, extending above the microtrichia and about 15–20 μm in length (Table 2). Trichoid sensilla are elongated and hair-shaped, arising from a thick base and gradually tapering to a pointed and slightly curved tip (Figure 4A,F,K,P,U and Figure 5A,G,L,P). Trichoid sensilla are the most common sensillar type on the postpedicel, especially in the middle and distal regions. The density of the trichoid sensilla increases from the proximal to the distal region, and more trichoid sensilla are present on the anterior surface (Table 3).

#### 3.2.2. Basiconic Sensilla

Basiconic sensilla (Ba) are tapered pegs that arise from slight depressions with dense micropores on the surface. Basiconic sensilla are usually shorter than trichoid sensilla (Table 2). Three subtypes of basiconic sensilla were identified according to their shape and size. Subtype I basiconic sensilla (Ba I) (Figure 4B,G,L,Q,V and Figure 5B,H,L,P) are generally thicker and longer, 7–10 μm in length, and have sharp tips at the end. Subtype II basiconic sensilla (Ba II) (Figure 4C,H,M,R,W and Figure 5C,I,M,Q) are generally thinner and shorter, 5–6 μm in length, and blunt-tipped. Subtype III basiconic sensilla (Ba III) are present on the postpedicel of *A*. *mihalyii*, shorter in length than Ba I and Ba II, and have a slightly thicker base (Figure 5D). Basiconic sensilla are only distributed in the middle and distal regions of the postpedicel and in a lower density compared to trichoid sensilla. Generally, the density of basiconic sensilla on the anterior surface is higher than that on the posterior surface (Table 3).

#### 3.2.3. Coeloconic Sensilla

Coeloconic sensilla (Co) are cone-shaped, characterized by longitudinally grooved walls that form a finger-like structure at their tips, and located in slight depressions on the surface of the postpedicel (Figure 4D,I,N,S,X and Figure 5E,J,N,R). Coeloconic sensilla are smaller in length and basal diameter compared to other types of sensilla, and they are about 2–4 μm in length (Table 2). Their distribution is relatively even on the surface of the postpedicel and is more sparse than the trichoid and basiconic sensilla. Typically, more coeloconic sensilla are found on the distal region of the postpedicel, with similar densities on the anterior and the posterior surfaces (Table 3).

#### 3.2.4. Clavate Sensilla

The clavate sensilla (Cl) are club-shaped, with a pointed tip and distal swelling. Their surface is characterized by micropores (Figure 4E,J,O,T,Y and Figure 5F,K,O,S). Clavate sensilla are of a similar length to subtype I basiconic sensilla (Table 2) but are different from other sensilla types, as they are only detected on the most proximal region of the postpedicel. Their density is higher on the anterior surface than on the posterior surface (Table 3).

#### 3.2.5. Sensory Pits

Several single-chambered recess structures, referred to as sensory pits (SP), are found on the postpedicel surface. They are mainly distributed in the proximal and middle regions of both the dorsal and ventral surfaces of the postpedicel (Figure 6). The sensory pits are more numerous in the females in comparison with the males (Table 1). Either coeloconic-like sensilla (Col) or bottle-shaped sensilla (BSS) cluster in each sensory pit. Several microtrichiae and coeloconic-like sensilla were observed in the sensory pits of *S*. *albiceps*, *S*. *peregrina*, *W*. *bella*, and *W*. *magnifica* (Figure 8). The coeloconic-like sensilla found in sensory pits resemble the coeloconic sensilla on the postpedicel surface but are longer (Table 2). Clusters of bottle-shaped (measuring 7–10 μm in length) sensilla could be observed on the inner surface of the sensory pits in all nine sarcophagid species (including females, Figure 7 and Figure 8). They are characterized by a basal swelling and gradually tapering tips; the micropores resemble chemoreceptor sensilla observed on the surface (Figure 7). Although the function of the basal swelling is unknown, different levels of swelling are found in different species (Table 2), from strongly swollen, almost round in *S*. *portschinskyi* and *A*. *mihalyii*, to slightly swollen in other species (Figure 7).

## 4. Discussion

In this study, we provide a comprehensive micromorphology of the antenna for nine species, including all three subfamilies of Sarcophagidae. Their morphological traits and sensilla types are generally similar to those of other Calyptratae species [30,45,46,48,49,50,51,52,53,54,55,56]. A total of eight types of antennal sensilla (chaetic sensilla, setiferous plaques, pedicellar buttons, trichoid sensilla, basiconic sensilla, coeloconic sensilla, clavate sensilla, bottle-shaped sensilla) and sensory pits are found in both sexes of the examined species. Where females are studied, they possess more sensory pits on the ventral and dorsal surfaces of the postpedicel than the males (*M*. *mesomelaena*, *W*. *bella*, *S*. *portschinskyi*).

The external morphology of the antennae within calyptrate flies are in general uniform, and yet a significant variation of sensory pits on the postpedicel is observed in different species [30,50,51,52]. Various types of sensilla, such as different types of basiconic sensilla [53], coeloconic sensilla [54], and microtrichiae, usually appear individually or in groups in the sensory pits. Nevertheless, what differentiates the sarcophagids from other flies is the bottle-shaped sensilla located in the sensory pits. The bottle-shaped sensilla were shown in a sarcophagid species (*S*. *carnaria*) by Smith and Lefroy [36] for the first time and were characterized by the portion resembling the neck of the bottle produced to a great extent. Later, the sensory pits of *S*. *argyrostoma* [37], *S*. *dux* [30], and *S*. *tibialis* [39] were also found to have bottle-shaped sensilla. Slifer and Sekhon [37] conducted an extensive TEM study on the antennal sensilla of *S*. *argyrostoma*. Neurons and dendrites inside the cuticular sheaths of the bottle-shaped sensilla increased the probability that they are olfactory organs. This unique sensilla type was not recorded in any other calyptrate flies (Supplementary File S1) but was common among all the species included in our study. Thus, this may be a potential new synapomorphy of Sarcophagidae, adding to the limited morphological characters that distinguish this large group of flies from other Calyptrates, although more studies should be conducted to confirm whether this type of sensilla is conserved among all sarcophagid flies.

Different levels of swelling in the bottle-shaped sensilla were observed in sarcophagid species (Table 2), from strongly swollen, almost round in *S*. *portschinskyi* and *A*. *mihalyii*, to less swollen in other examined species (Figure 7). Sensory organs on the postpedicel play vital roles in locating mates, foods, habitats, hosts, and oviposition sites. The larvae of *A*. *mihalyii* are mainly parasitoids of the last instar larvae and pupae of Lepidoptera, also attacking sawflies on some occasions [15,16,17]. The level of swelling in the bottle-shaped sensilla observed in *A*. *mihalyii* may be associated with host localization. The swollen bottle-shaped sensilla may be able to accommodate more neurons and dendrites, which could facilitate odor detection. Further electrophysiological and neurological studies are needed to elucidate the function of the bottle-shaped sensilla and why they evolved in sarcophagid flies.

Three morphologically different subtypes of chaetic sensilla were detected on the pedicel. The nonporous surface and the presence of a socket at the base of sensilla chaetica suggest that they have a mechanosensory function. Several setiferous plaques containing bulbous setae were found on the pedicel. The arrangement and number of the setiferous plaques are similar to those reported for other sarcophagid species [30,39]. Different from the long and tapered setae of plaques in Fanniidae and Muscidae [42] and the short and apically obtuse setae of plaques in Scathophagidae [55] and Anthomyiidae [57], the plaques of all the examined flesh flies possess a short seta with a sharply abrupt tip. In some of the specimens examined, some of the bulbous setae were missing. A loss of bulbous setae in some plaques is always detected [30,42]. Greenberg [58] first found the setiferous plaque as a new structure on fly antennae and mentioned that the bulbous setae are lost with age. After the separation of antennal pedicel and postpedicel, a pedicellar button with a circular central dome could be found in all the examined species, which has first been addressed in the family Sarcophagidae. Unlike the poreless pedicellar buttons discovered in Calyptratae (e.g., Oestridae [49]; Tachinidae [54]; Anthomyiidae [57]), these structures display one micropore at the center of the pedicellar button in *S. albiceps*, *S*. *africa*, *S*. *peregrina*, and *S*. *portschinskyi*. The presence of a micropore on the pedicellar button may indicate a potential chemoreceptory function [48]. 

Interestingly, the morphology of each sensilla type on the postpedicel, i.e., trichoid sensilla, basiconic sensilla, coeloconic sensilla, and clavate sensilla, usually varies within a small range among the nine species of sarcophagid flies (Table 2). This hierarchical sensilla arrangement is found in several other groups of flies such as Oestridae and in the genus *Lispe* Latreille (Muscidae) [49,59]. In fact, the size of the same type of sensilla remains largely similar in flies despite the significant differences in body size, antennal morphology, olfactory preference, and biology. This sensillar size variation may be related to capturing the olfactory cues of different physical properties as they pass the antennal surface in the airflow [28]. Further investigations are needed to verify this hypothesis.

Sensory pits may play a central role in the olfactory function of sarcophagid flies. Compared with other insect groups with a long postpedicel (e.g., Coleoptera; Hymenoptera; Lepidoptera), calyptrate flies usually have smaller antennae and fewer sensilla [27]. A unique character of this group is that a large proportion of the sensilla are concealed in the cuticular depressions (sensory pits or inner sacculus), which a priori would seem to limit the chances for the sensilla to capture odor molecules in the air, thereby reducing the capability of odor detection. However, field observations have shown that blowflies and flesh flies are attracted by compounds released at the early stage of decomposition and that they arrive at carcasses much earlier than other necrophagous animals [56,60,61]. In order to gain advantages in locating corpses and hosts as early as possible, flesh flies must be equipped with excellent sensory organs that can detect the faintest odors. Sukontason et al. [30] conducted a comparison of the antennal sensilla among a blowfly, a muscid fly, and a flesh fly and found the latter to bear more sensory pits. Among the nine species in this study, the number of sensory pits of parasitoids and parasites (*A*. *mihalyii*, *W*. *magnifica*) is approximately equal to the necrophages (*Sarcophaga* spp., *M*. *mesomelaena*) and agents of myiasis (*W*. *magnifica*), and higher than for kleptoparasites (*Metopia campestris*), demonstrating the significance of sensory pits in odor detection [39,62,63].

## 5. Conclusions

In conclusion, the detailed antennal ultrastructure, morphology, and density of each antennal sensilla of nine sarcophagid species were provided. The bottle-shaped sensilla with pores were found in the sensory pit of the antennal postpedicel in members of all three flesh fly subfamilies, providing a potential new synapomorphy for the family Sarcophagidae. We suggest that the concentration of the same type of sensilla may allow access for more chemicals to the dendrites of chemosensory neurons inside the sensory pit. Even though molecular techniques are commonly used in insect phylogenetics today, our research also sheds light on the need to consider the antennal ultrastructure in exploring the phylogeny and evolutionary adaptions of flesh flies.

## Figures and Tables

**Table 1:** The number of sensory pits on the dorsal and ventral surfaces of the postpedicel of nine examined species. (− = undetermined, *n* = 1).

Species	Number of Sensory Pits (Ventral Surface)		Number of Sensory Pits (Dorsal Surface)	
	Male	Female	Male	Female
* Sarcophaga albiceps *	12	−	4	−
* Sarcophaga africa *	10	−	6	−
* Sarcophaga peregrina *	6	−	4	−
* Sarcophaga portschinskyi *	10	15	6	7
* Agria mihalyii *	14	−	11	−
* Wohlfahrtia magnifica *	1	−	1	−
* Wohlfahrtia bella *	6	10	2	16
* Metopia campestris *	3	−	1	−
*Mesomelena mesomelaena*	2	8	0	26

**Table 2:** Length, basal diameter, and degree of basal swelling (maximum diameter of the ‘neck’ of the bottle/maximum diameter of the bottle half) of the sensilla on the antennal postpedicel of nine male sarcophagid species (μm ± SD, *n* = 10). Abbreviations: Ba I (basiconic sensilla I); Ba II (basiconic sensilla II); Ba III (basiconic sensilla III); BSS (bottle-shaped sensilla); Cl (clavate sensilla); Co (coeloconic sensilla); Col (coeloconic-like sensilla); Tr (trichoid sensilla); − (undetermined).

Species	Type	Length	Basal Diameter	Degree of Basal Swelling
* Sarcophaga albiceps *	Tr	19.64 ± 2.14	3.07 ± 0.35	−
	Ba I	7.77 ± 0.84	1.94 ± 0.30	−
	Ba II	5.50 ± 0.28	1.04 ± 0.17	−
	Co	3.39 ± 0.66	0.98 ± 0.39	−
	Col	5.85 ± 0.48	1.98 ± 0.41	−
	Cl	7.88 ± 0.66	1.72 ± 0.24	−
	BSS	7.02 ± 0.38	1.76 ± 0.22	0.51 ± 0.03
*Sarcophaga portschinskyi*	Tr	15.71 ± 1.24	2.51 ± 0.37	−
	Ba I	9.01 ± 0.93	1.81 ± 0.25	−
	Ba II	6.43 ± 0.88	1.85 ± 0.32	−
	Co	3.45 ± 0.84	1.06 ± 0.25	−
	Cl	9.35 ± 1.18	1.85 ± 0.25	−
	BSS	7.69 ± 1.05	2.46 ± 0.55	0.41 ± 0.01
*Sarcophaga africa*	Tr	18.30 ± 1.43	2.18 ± 0.27	−
	Ba I	7.58 ± 0.40	1.87 ± 0.42	−
	Ba II	6.88 ± 0.56	1.28 ± 0.04	−
	Co	3.92 ± 0.53	1.35 ± 0.19	−
	Cl	6.80 ± 0.40	1.33 ± 0.42	−
	BSS	8.02 ± 0.69	1.81 ± 0.30	0.35 ± 0.01
*Sarcophaga peregrina*	Tr	21.02 ± 3.43	2.20 ± 0.41	−
	Ba I	9.36 ± 1.43	1.37 ± 0.46	−
	Ba II	6.00 ± 0.82	1.53 ± 0.12	−
	Co	3.41 ± 0.42	1.02 ± 0.09	−
	Col	8.30 ± 0.32	1.72 ± 0.21	−
	Cl	8.64 ± 0.96	1.87 ± 0.20	−
	BSS	9.42 ± 1.00	2.15 ± 0.23	0.21 ± 0.02
*Wohlfahrtia bella*	Tr	18.23 ± 2.4	2.30 ± 0.26	−
	Ba I	10.15 ± 0.54	1.45 ± 0.25	−
	Ba II	6.57 ± 0.21	1.74 ± 0.56	−
	Co	3.04 ± 1.13	1.25 ± 0.22	−
	Cl	9.53 ± 0.82	1.56 ± 0.25	−
	BSS	11.28 ± 1.31	2.32 ± 0.42	0.25 ± 0.01
*Wohlfahrtia magnifica*	Tr	16.51 ± 1.64	2.47 ± 0.15	−
	Ba I	10.12 ± 2.51	1.90 ± 0.65	−
	Ba II	5.84 ± 0.52	1.20 ± 0.32	−
	Co	3.48 ± 0.04	0.75 ± 0.02	−
	Col	4.57 ± 0.44	1.17 ± 0.01	−
	Cl	11.30 ± 1.32	1.70 ± 0.14	−
	BSS	9.29 ± 0.60	1.74 ± 0.14	0.34 ± 0.01
*Agria mihalyii*	Tr	19.16 ± 1.49	2.90 ± 0.26	−
	Ba I	7.70 ± 1.18	1.34 ± 0.38	−
	Ba II	6.00 ± 0.23	1.82 ± 0.77	−
	Ba III	4.86 ± 0.09	2.20 ± 0.81	−
	Co	2.93 ± 0.30	1.16 ± 0.05	−
	Cl	8.62 ± 0.66	1.68 ± 0.36	−
	BSS	7.89 ± 1.02	4.80 ± 0.84	0.09 ± 0.01
*Metopia campestris*	Tr	19.07 ± 2.48	2.33 ± 0.48	−
	Ba I	9.07 ± 1.44	1.50 ± 0.08	−
	Ba II	5.50 ± 0.45	1.35 ± 0.22	−
	Co	3.44 ± 0.49	1.09 ± 0.12	−
	Cl	8.90 ± 0.99	1.35 ± 0.43	−
	BSS	6.43 ± 0.27	1.81 ± 0.08	0.42 ± 0.02
*Mesomelena mesomelaena*	Tr	20.05 ± 2.56	2.50 ± 0.56	−
	Ba I	10.25 ± 1.23	1.50 ± 0.03	−
	Ba II	6.70 ± 1.62	1.25 ± 0.20	−
	Co	3.45 ± 0.24	1.20 ± 0.14	−
	Cl	10.05 ± 0.24	1.58 ± 0.36	−
	BSS	10.64 ± 0.45	2.05 ± 0.32	0.23 ± 0.01

**Table 3:** Average density of sensilla on the antennal postpedicel of nine male sarcophagid species (10^−3^ μm^−2^ ± SD, *n* = 20). Abbreviations: Ba (basiconic sensilla); Cl (clavate sensilla); Co (coeloconic sensilla); Tr (trichoid sensilla). The proximal, median, and distal regions each consist of one-third of the antenna in length.

Species	Type	Anterior Surface			Posterior Surface		
		Proximal	Median	Distal	Proximal	Median	Distal
*Sarcophaga. albiceps*	Tr	4.60 ± 2.51	13.00 ± 1.92	13.60 ± 1.14	1.00 ± 1.00	10.00 ± 2.92	11.40 ± 3.36
	Ba	0.00	11.40 ± 1.67	6.20 ± 1.79	0.00	9.20 ± 0.84	8.00 ± 2.12
	Co	0.00	0.00	1.40 ± 0.55	0.20 ± 0.45	3.00 ± 1.41	2.80 ± 0.84
	Cl	7.00 ± 1.22	0.00	0.00	4.20 ± 1.10	0.00	0.00
*Sarcophaga portschinskyi*	Tr	2.80 ± 2.77	14.00 ± 4.53	22.20 ± 0.84	0.00	6.60 ± 2.19	13.40 ± 1.95
	Ba	0.00	9.40 ± 2.07	8.60 ± 4.67	0.00	4.80 ± 1.64	5.20 ± 0.84
	Co	0.20 ± 0.45	0.00	4.00 ± 2.55	0.20 ± 0.45	0.20 ± 0.45	1.60 ± 1.34
	Cl	7.60 ± 2.30	0.00	0.00	5.00 ± 1.22	0.00	0.00
*Sarcophaga africa*	Tr	9.80 ± 6.26	12.00 ± 2.74	20.80 ± 4.02	1.00 ± 1.00	4.00 ± 2.55	16.80 ± 2.17
	Ba	0.00	12.20 ± 1.79	7.60 ± 1.52	0.00	4.00 ± 1.22	4.40 ± 1.14
	Co	0.20 ± 0.45	0.20 ± 0.45	2.60 ± 1.52	0.40 ± 0.89	0.00	2.20 ± 1.30
	Cl	7.20 ± 1.79	0.00	0.00	6.00 ± 1.00	0.00	0.00
*Sarcophaga peregrina*	Tr	9.20 ± 1.48	9.80 ± 1.92	18.20 ± 3.63	0.00	4.20 ± 1.92	12.80 ± 2.49
	Ba	0.00	7.60 ± 2.07	6.40 ± 2.30	0.00	7.00 ± 2.92	6.80 ± 4.60
	Co	0.80 ± 0.84	1.60 ± 1.14	2.60 ± 0.55	1.00 ± 0.71	1.20 ± 1.30	2.20 ± 1.79
	Cl	2.00 ± 1.58	0.00	0.00	7.00 ± 2.35	0.00	0.00
*Wohlfahrtia bella*	Tr	4.25 ± 1.56	6.20 ± 3.25	21.00 ± 2.45	1.00 ± 1.24	5.60 ± 2.12	15.02 ± 1.67
	Ba	0.00	3.50 ± 0.75	2.40 ± 1.63	0.00	1.20 ± 0.40	1.40 ± 1.45
	Co	0.00	0.40 ± 1.20	0.30 ± 0.54	0.00	0.00	0.20 ± 0.45
	Cl	1.50 ± 0.30	0.00	0.00	0.60 ± 0.25	0.00	0.00
*Wohlfahrtia magnifica*	Tr	2.27 ± 2.85	5.33 ± 2.88	13.61 ± 4.54	1.81 ± 1.89	3.33 ± 5.41	13.04 ± 3.52
	Ba	0.00	0.11 ± 0.51	0.79 ± 1.33	0.00	0.30 ± 0.80	1.13 ± 1.73
	Co	0.00	0.23 ± 0.70	0.23 ± 0.70	0.00	0.00	0.23 ± 0.70
	Cl	0.23 ± 0.70	0.00	0.00	0.45 ± 1.19	0.00	0.00
*Agria mihalyii*	Tr	0.40 ± 0.55	7.00 ± 1.58	19.00 ± 4.24	0.20 ± 0.45	3.80 ± 1.64	17.20 ± 5.22
	Ba	0.00	3.20 ± 1.79	6.00 ± 3.32	0.00	0.80 ± 1.30	7.00 ± 1.87
	Co	0.00	0.20 ± 0.45	1.60 ± 1.14	0.00	0.20 ± 0.45	1.20 ± 1.30
	Cl	1.60 ± 1.52	0.00	0.00	0.60 ± 0.55	0.00	0.00
*Metopia campestris*	Tr	18.20 ± 7.19	30.40 ± 3.78	35.60 ± 3.51	14.00 ± 7.91	25.40 ± 6.88	34.00 ± 4.85
	Ba	0.00	3.20 ± 1.64	2.00 ± 1.87	0.00	3.60 ± 0.89	1.40 ± 0.89
	Co	0.00	1.20 ± 1.30	2.00 ± 1.41	0.00	1.40 ± 1.52	2.00 ± 1.41
	Cl	3.00 ± 2.12	0.00	0.00	1.60 ± 1.34	0.00	0.00
*Mesomelena mesomelaena*	Tr	17.50 ± 3.52	28.60 ± 2.63	32.20 ± 0.51	9.20 ± 3.62	22.30 ± 2.74	30.60 ± 0.62
	Ba	0.00	2.54 ± 0.73	4.00 ± 2.43	0.00	2.40 ± 0.65	2.20 ± 1.32
	Co	0.00	2.40 ± 0.86	1.60 ± 0.13	0.00	0.45 ± 3.41	1.30 ± 0.70
	Cl	3.20 ± 0.23	0.00	0.00	0.00	0.00	0.00

## Data Availability

The data generated in this study are provided here, and they are also available upon request from the corresponding author.

## Supplementary Materials

The following supporting information can be downloaded at: https://www.mdpi.com/article/10.3390/insects13070602/s1, Supplementary File S1. Comparison of antennal sensilla on the antennal postpedicel surface of sarcophagid flies and other calyptrate flies [64,65,66,67,68,69,70,71,72,73,74,75,76,77,78,79,80,81].
