# Supplementary material for: Ultrastructure of Antennal Sensory Organs in Nine Flesh Flies (Diptera: Sarcophagidae): New Insight into the Definition of Family Sarcophagidae
Source: Insects. 2022 Jun 30;13(7):602. doi: 10.3390/insects13070602 (PMC9316071; doi:10.3390/insects13070602)
Supplement: Supplementary file 1 [file insects-13-00602-s001.zip › insects-1780296-SI.pdf]

Article

Ultrastructure of Antennal Sensory Organs in Nine Flesh Flies (Diptera: Sarcophagidae): New Insight into the Definition of Family Sarcophagidae

Wentian Xu [1], Genting Liu [1,2], Qike Wang [2], Liping Yan [1], Xianhui Liu [3], Xinyu Li [1], Thomas Pape [4], and Dong Zhang [1,*]

[1] School of Ecology and Nature Conservation, Beijing Forestry University, Qinghua East Road 35,
Beijing 100083, China; xuwt720@bjfu.edu.cn (W.X.);
gentingl@student.unimelb.edu.au (G.L.);
yanlp@bjfu.edu.cn (L.Y.); lixinyu91@cau.edu.cn (X.L.)

[2] School of BioSciences, University of Melbourne, Melbourne, VIC 3010, Australia; wangqike123@gmail.com

[3] Department of Entomology and Nematology, University of California Davis, Davis, CA 95616, USA; xhfliu@ucdavis.edu

[4] Natural History Museum of Denmark, Science Faculty, University of Copenhagen, Copenhagen, 2100, Denmark; tpape@snm.ku.dk

* Correspondence: zhangdong_bjfu@bjfu.edu.cn

Supplementary File S1

Comparison of antennal sensilla on the antennal postpedicel surface of sarcophagid flies and other Calyptrate flies.

BSS = bottle-shaped sensilla; SP = sensory pit; √ = identified; × = not identified; − = no data.

Super family	Family	Subfamily	Species	SP	BSS	Reference
**Hippoboscoidea**	Glossinidae		*Glossina palpalis*	√	×	64
			Glossina tachinoides	√	×	
	Hippoboscidae		*Lipoptena cervi*	×	×	52
			Lipoptena fortisetosa	×	×	
			Melophagus ovinus	√	×	
			Hippobosca longipennis	√	×	65
			Hippobosca equina	√	×	
**Muscoid grade**	Fanniidae		*Fannia hirticeps*	√	×	51
			Fannia scalaris	√	×	46
			Fannia canicularis	√	×	
	Muscidae		*Musca domestica*	√	×	45
			Hydrotaea irritans	√	×	66
			Hydrotaea chalcogaster	√	×	67
			Hydrotaea armipes	√	×	32
			Ophyra capensis	√	×	68
			Lispe neimongola	√	×	25
	Anthomyiidae		*Delia radicum*	√	×	69
			Delia platura	√	×	
			Delia floralis	√	×	29
			Delia antiqua	√	×	
	Scathophagidae		*Scathophaga stercoraria*	√	×	55
**Oestriodea**	Sarcophagidae	Sarcophaginae	*Sarcophaga albiceps*	√	√	Present study
			Parasarcophaga dux	√	−	30
			Sarcophaga portschinskyi	√	√	Present study
			Sarcophaga africa	√	√	Present study
			Sarcophaga peregrina	√	√	Present study
			Sarcophaga carnaria	√	√	36
			Sarcophaga argyrostoma	√	√	37
			Sarcophaga tibialis	√	√	39
		Paramacronychiinae	*Wohlfahrtia bella*	√	√	Present study
			Wohlfahrtia magnifica	√	√	Present study
			Agria mihalyii	√	√	Present study
		Miltogramminae	*Metopia campestris*	√	√	Present study
			Mesomelena mesomelaena	√	√	Present study
	Calliphoridae	Calliphorinae	*Lucilia sericata*	√	×	48
			Lucilia cuprina	√	×	70
			Phormia regina	√	×	71
			Protophormia terraenovae	√	×	72
			Hemipyrellia ligurriens	√	×	73

		Chrysomya megacephala	√	×	
		Chrysomya rufifacies	√	×	74
		Chrysomya nigripes	√	×	
		Chrysomya pinguis	√	×	74
		Triceratopyga calliphoroides	√	×	75
Mesembrinellidae		*Mesembrinella bellardiana*	√	–	
		Mesembrinella bicolor	√	–	76
		Mesembrinella semihyalina	√	–	
Tachinidae	Exoristinae	*Pales pavida*	√	×	54
		Pseudoperichaeta nigrolineatu	√	×	77
		Gymnosoma rotundatum	√	×	78
	Phasiinae	*Trichopoda pennipes* F.	√	×	79
Oestridae	Oestrinae	*Rhinoestrus purpureus*	√	×	53
		Oestrus ovis	√	×	80
	Hypodermatinae	*Hypoderma lineatum*	√	×	81
		Hypoderma bovis	√	×	50
		Portschinskia magnifica	×	×	24
	Gasterophilinae	*Gasterophilus nigricornis*	√	×	23
		Gasterophilus pecorum	√	×	49
		Gasterophilus nasalis	√	×	49
		Gasterophilus intestinalis	√	×	49
		Gasterophilus haemorrhoidalis	√	×	49
